# Supplementary material for: Prevalence of Everyday Discrimination and Relation with Wellbeing among Aboriginal and Torres Strait Islander Adults in Australia
Source: Int J Environ Res Public Health. 2021 Jun 18;18(12):6577. doi: 10.3390/ijerph18126577 (PMC8296443; doi:10.3390/ijerph18126577)
Supplement: Supplementary file 1 [file ijerph-18-06577-s001.zip › ijerph-1247833-supplementary.pdf]

ADDITIONAL FILE 1

Table S1. Detailed variable definitions

Variables	
**Discrimination and attribution**	
**Discrimination in everyday life (Q95)**	Participants are asked, "How often do these things happen to you?" [note: no time window provided] with the response options, "not at all" (1), "a little bit" (2), "a fair bit" (3), or "a lot" (4). These are the response options used for many questionnaire items on the MK survey, as they were determined to be readily understood/acceptable response options through the focus group process.<br><br>There are 8 prompts:<br>1. I am treated with less respect than other people.<br>2. I receive worse service than other people (including at restaurants, stores, Centrelink, housing).<br>3. People act like I am not smart.<br>4. People act like they are afraid of me.<br>5. I am called names, insulted, or yelled at.<br>6. I am followed around in shops.<br>7. I am watched more closely than others at work or school.<br>8. Police unfairly bother me.<br><br>Items are coded as "." if missing data.<br><br>For each item, response options included "not at all" (coded as 0), "a little bit" (1), "a fair bit" (2), and "a lot" (3). The total score is calculated by summing responses to all 8 items (range: 0 to 24); the total score is only created for participants with complete data across the items. In the validation study, responses were categorised as no (score:0/24), low (1-8/24), moderate (9-16/24), or high discrimination (17-24/24). For this analysis, the moderate and high categories were combined to avoid small cells.<br><br>Note: Participants with missing data on the total score are excluded from the current analysis.
**Attribution of discrimination to Indigenous status (Q96, Q99)**	After the instrument (i.e. after the 8 items), participants are asked "When these things happen, do you think it is because you are Aboriginal/Torres Strait Islander?", with the response options, "not at all" (1), "a little bit" (2), "a fair bit" (3), or "a lot" (4).<br><br>Participants were categorised as experiencing no discrimination (based on total score, coded as 0), or coded as experiencing any discrimination attributed to Indigeneity "not at all" (coded as 1), "a little bit" (2), "a fair bit" (3), or "a lot" (4).<br><br>This is also collapsed as: experiencing no discrimination (based on total score, coded as 0), or coded as experiencing any discrimination with *no* attribution to Indigeneity (coded as 1) or with *any* attribution to Indigeneity (coded as 2, including responses of "a little bit, "a fair bit", and "a lot").
**Discrimination score *X* attribution**	Variable combining total discrimination score with attribution (categorised as no discrimination, low discrimination without attribution, low discrimination with attribution, moderate/high discrimination without attribution, moderate/high discrimination with attribution).
**Covariates**	
**Age group**	Age is analysed as a categorical variable, categorised as: 16-35 years, 36-55 years, ≥56 years; this broad grouping is used to avoid small/zero cells. Where a further collapsed age category was required, the following categorisation was used: 16-65 years, ≥66 years.
**Gender**	Coded as (1) male, (2) female, (3) other, (.) missing.<br><br>There are a small number of persons identifying as other gender. Therefore, we cannot present data for this group on their own, and we cannot include this category when gender is used as an adjustment variable. A variable including those identifying as males and females only is used for all stratified and adjusted analyses: (1) male (2) female, (.) missing/other. However, participants identifying as another gender are included in analyses of the overall sample and any analyses that are not adjusted for or stratified by gender.
**Level of remoteness**	Remoteness is based on self-reported postcode of home address, and categorised as major city, inner and outer regional area, or remote and very remote area.

**State/Territory**	State/Territory of residence was the Australian Capital Territory (ACT), New South Wales (NSW), Northern Territory (NT), Queensland (QLD), South Australia (SA), Tasmania (TAS), Victoria (VIC), or Western Australia (WA).
**Highest educational qualification**	Participants were asked, “What is the highest education you have completed?” with the response options of: no school, primary school, some high school – categorised as (1) less than Year 10; Year 10 (School or Intermediate certificate) – categorised as (2) Year 10; Year 12 (Higher school, leaving certificate, College), Certificate or diploma (such as child care worker, mechanic) – categorised as Year 12 or beyond.
**Family financial situation**	Participants were asked, “Which words best describe your family's money situation?” with response options of: We have a lot of savings, We have some savings – categorised as (1) We have some or a lot of savings; We have just enough to get us to the next payday – categorised as (2) We have just enough; We run out of money before payday, We are spending more than we get – categorised as (3) We run out of money or spend more than we get; Unsure – categorised as (4) Unsure.
**Outcome measures**	
**SEWB outcomes**	
**Pain**	Participants are asked “In the last 4 weeks about how often did you feel pain?”, with the response options: (1) all of the time (2) most of the time (3) some of the time (4) a little of the time (5) none of the time.<br><br>Collapsed and coded as (0) none or a little bit of the time (1) some to all of the time (frequent experience of pain).<br><br>Preliminary validation work of this item,[1] including responses to this survey item and the follow-up free-text question, “If yes, what type of pain?” demonstrates that the measure is capturing holistic experience of pain, including physical pain but also pain related to disconnection from country, culture, family, and community. Therefore it is considered a measure of SEWB.
**Life satisfaction**	Participants are asked “How satisfied are you with your life?”, with the response options: (1) a lot (2) a fair bit (3) a little bit (4) not at all.<br><br>Collapsed and coded as (0) A fair bit to a lot (1) not at all or a little bit (low life satisfaction).
**Happiness**	Participants are asked “In the last 4 weeks how often did you feel happy?” , with the response options: (1) all of the time (2) most of the time (3) some of the time (4) a little of the time (5) none of the time.<br><br>Collapsed and coded as (0) some to all of the time (1) none or a little bit of the time (low happiness).
**Psychological distress (Q50)**	Psychological distress is measured using a modified Kessler-5 scale (MK-K5). Participants are asked, “In the last 4 weeks about how often did you …” for a set of prompts, with the response options “All of the time” (5), “Most of the time” (4), “Some of the time” (3), “A little of the time” (2), or “None of the time” (1). This is the same response option and response ordering as used in the ABS survey. Preliminary validation work has demonstrated the validity of this measure for this scale.[2]<br><br>There are 8 prompts; bolded prompts indicate the MK-K5 questions (text in square brackets indicates the original K5 wording):<br>1. … feel happy?<br>2. … feel worried?<br>**3. … feel nervous?**<br>**4. … feel hopeless (have no hope)?** [K5 wording: “without hope”]<br>**5. … feel restless or jumpy?**<br>**6. … feel everything was an effort (have no energy)?** [K5 wording does not include clarifier (have no energy)]<br>**7. … feel sad?** [K5 wording: “so sad that nothing could cheer you up?”]<br>8. … feel pain? (If yes, what kind of pain:_______)<br><br>Individual items are named: k5_nervous k5_hopeless k5_restless k5_effort k5_sad.<br><br>The MK-K5 total score is a sum of the 5 MK-K5items, range: 5-25. MK-K5total score is missing if any of the individual items were missing.<br><br>A categorical MK-K5 variable is created based on established commonly-used K-5 cut-offs (e.g. used in NATSIHS): Low: 5–7.99 (0); Moderate 8–11.99 (1); High: 12–14.99 (2); Very High: 15– 25 (3).

	A binary MK-K5 “high/very high” distress variable is created: Low or moderate distress (5-<12) (0); High or very high psychological distress (12-25) (1).
**Doctor reported anxiety**	Participants were asked to state if a doctor had ever told them that they had a range of health-related conditions. For each condition, participants ticked the box if they had ever had the condition. Those who did not tick the box are coded as 0, noting that this represents participants who answered no as well as participants who did not answer the question – it is not possible to distinguish between the two.<br><br>Anxiety: Coded as (0) not selected (1) selected.
**Doctor reported depression**	Participants were asked to state if a doctor had ever told them that they had a range of health-related conditions. For each condition, participants ticked the box if they had ever had the condition. Those who did not tick the box are coded as 0, noting that this represents participants who answered no as well as participants who did not answer the question – it is not possible to distinguish between the two.<br><br>Depression: Coded as (0) not selected (1) selected.
**Cultural outcomes**	
**Control over life**	Participants asked, “How much are you in control of your life?”, with the response options: (1) a lot (2) a fair bit (3) a little bit (4) not at all.<br><br>Collapsed and coded as (0) A fair bit to a lot (1) not at all to a little bit.<br><br>This measure of personal control is included as an indicator of self-determination, which is a key cultural determinant for Aboriginal and Torres Strait Islander peoples. Previous research has demonstrated that control is linked to colonisation and the historical context.[3,4] In the absence of a validated measure of personal control for Aboriginal and Torres Strait Islander peoples,[4] this single item is used in the current study.
**Feeling torn between Aboriginal and Torres Strait Islander and non-Indigenous culture**	Participants asked “Do you feel torn between your culture and non-Indigenous culture?”, with the response options: (1) not at all (2) a little bit (3) a fair bit (4) a lot (5) unsure.<br><br>Collapsed and coded as (0) not at all (1) a little or fair bit (2) a lot (3) unsure.<br><br>Binary variable created (0) not at all (1) a little bit to a lot (feel torn between cultures). For the binary variable, responses of “unsure” are recoded to missing.
**Feeling disconnected from Aboriginal and Torres Strait Islander culture**	Participants are asked “have you ever felt disconnected from Aboriginal/Torres Strait Islander culture?”, with the response options: (1) not at all (2) a little bit (3) a fair bit (4) a lot (5) unsure.<br><br>Collapsed and coded as (0) not at all (1) a little or fair bit (2) a lot (3) unsure (.) missing.<br><br>Binary variable created (0) not at all (1) a little bit to a lot. For the binary variable, responses of “unsure” are recoded to missing.
**Choose not to identify as Aboriginal and Torres Strait Islander in the Census, study, work, Centrelink, or housing**	Participants were asked to state if they chose *not* to identify as Aboriginal and/or Torres Strait Islander in a range of contexts. For each context, participants ticked the box if they chose not to identify (coded as 1). Those who did not tick the box are coded as 0, noting that this represents participants who answered no as well as participants who did not answer the question – it is not possible to distinguish between the two.<br><br>We examined responses to the following contexts: Census, study, work, Centrelink, and housing.<br><br>Variables:<br>• Census: (0) not selected (1) do not self-identify on Census<br>• Study: (0) not selected (1) do not self-identify at School or Uni<br>• Work: (0) not selected (1) do not self-identify at work<br>• Centrelink: (0) not selected (1) do not self-identify with Centrelink<br>• Housing: (0) not selected (1) do not self-identify with housing/real estate<br><br>Overall binary variable capturing participants reported that they did not self-identify as Aboriginal and/or Torres Strait Islander in *one or more of these contexts*: (0) not selected (1) do not self-identify for one or more.<br><br>For each context, participants ticked the box if they chose not to identity in that context. Those who did not tick the box are coded as 0, noting that this represents participants

	who answered no as well as participants who did not answer the question – it is not possible to distinguish between the two.
**Health behaviours**	
**Current smoker**	Participants asked “Do you smoke?”, with the response options: (1) Yes, I currently smoke (2) I smoked in the past but don’t smoke now (3) I have never been a smoker.<br><br>Binary variable created, coded as (0) not current smoker (includes past and never smokers) (1) current smoker.
**Gamble**	Participants asked “Have you bet or spent money gambling in the last year?” (0) no (1) yes.
**Alcohol use**	Simply looking at current alcohol use may be misleading, because current non-drinkers are often past heavy drinkers who quit due to dependence or due to illness. Examining ever drinking (i.e. current or past drinking) has limited meaning because the vast majority of the sample has ever drunk alcohol. Instead, we focus on a measure of ever alcohol dependence. We note that there is no validated measure of alcohol dependence for the Aboriginal and Torres Strait Islander population.[5] In the absence of a validated measure of alcohol dependence, we used the following approach to define ever alcohol dependence.<br><br>Participants were asked, “do you drink alcohol?”, with the response options of (1) Yes (2) I drank in the past, but don’t drink now (3) I have never been a drinker.<br>• Those responding (1) current drinker are asked: “Do you get shaky if you don't have alcohol?”, with the response options of (0) No (1) Yes.<br>• Those responding (2) are asked: “When you used to drink, did you get shaky if you didn't have alcohol?”, with the response options of (0) No (1) Yes.<br><br>According to responses to these items, participants were categorised as follows:<br>0. Never drinker – responded (3) never drinker. Any responses to the question about currently/previously being “shaky” are disregarded as never drinkers should not have answered those questions.<br>1. Current or past drinker, never reported dependence<br>a. Includes those who responded (1) current drinker and responded (0) No don’t get shaky if don’t drink. Any response to the question about “used to get shaky” is disregarded as current drinkers should not have answered the question about past drinking.<br>b. Includes those who responded (2) past drinker and responded (0) No didn’t get shaky in the past if didn’t drink. Any response to the question about “currently get shaky” is disregarded as past drinkers should not have answered the question about current drinking.<br>2. Current or past drinker, ever reported dependence<br>a. Includes those who responded (1) current drinker and responded (1) Yes get shaky if don’t drink. Any response to the question about “used to get shaky” is disregarded as current drinkers should not have answered the question about past drinking.<br>b. Includes those who responded (2) past drinker and responded (1) Yes when used to drink, got shaky if didn’t drink. Any response to the question about “currently get shaky” is disregarded as past drinkers should not have answered the question about current drinking.<br>3. Missing (.)<br>a. Missing data on current drinking status and/or current shaky status (if a current drinker) and/or past shaky status (if a past drinker).
**Physical health**	
**General health**	Participants asked to self-rate their general health, from the response options of: (1) excellent (2) very good (3) good (4) fair (5) poor.<br><br>Collapsed and coded as (0) good to excellent (1) Fair or poor.
**Doctor reported heart disease**	Participants were asked to state if a doctor had ever told them that they had a range of health-related conditions. For each condition, participants ticked the box if they had ever had the condition. Those who did not tick the box are coded as 0, noting that this represents participants who answered no as well as participants who did not answer the question – it is not possible to distinguish between the two.<br><br>Heart disease: Coded as (0) not selected (1) selected.
**Doctor reported high blood pressure**	Participants were asked to state if a doctor had ever told them that they had a range of health-related conditions. For each condition, participants ticked the box if they had ever

	had the condition. Those who did not tick the box are coded as 0, noting that this represents participants who answered no as well as participants who did not answer the question – it is not possible to distinguish between the two.<br><br>High blood pressure: Coded as (0) not selected (1) selected.
**Doctor reported high cholesterol**	Participants were asked to state if a doctor had ever told them that they had a range of health-related conditions. For each condition, participants ticked the box if they had ever had the condition. Those who did not tick the box are coded as 0, noting that this represents participants who answered no as well as participants who did not answer the question – it is not possible to distinguish between the two.<br><br>High cholesterol: Coded as (0) not selected (1) selected.
**Doctor reported diabetes**	Participants were asked to state if a doctor had ever told them that they had a range of health-related conditions. For each condition, participants ticked the box if they had ever had the condition. Those who did not tick the box are coded as 0, noting that this represents participants who answered no as well as participants who did not answer the question – it is not possible to distinguish between the two.<br><br>Diabetes: Coded as (0) not selected (1) selected.

Table S2. Relationship between experiences of everyday discrimination and wellbeing outcomes (N=up to 8100)

	% (n) with outcome	Unadjusted PR (95%CI)	Adjusted PR (95%CI)
**SEWB OUTCOMES**			
**Outcome: frequent experience of pain (N=7817)* †**			
No discrimination	37.4 (1210)	1 (Ref)	1 (Ref)
Low discrimination	46.5 (1732)	1.24 (1.26,1.41)	1.33 (1.26,1.41)
Moderate/high discrimination	54.8 (466)	1.47 (1.53,1.77)	1.64 (1.53,1.77)
**Outcome: low life satisfaction (N=7931)* †**			
No discrimination	15.0 (495)	1 (Ref)	1 (Ref)
Low discrimination	30.9 (1166)	2.06 (1.84,2.23)	2.02 (1.84,2.23)
Moderate/high discrimination	52.9 (460)	3.52 (3.10,3.82)	3.44 (3.10,3.82)
**Outcome: low happiness (N=7952)* †**			
No discrimination	6.4 (210)	1 (Ref)	1 (Ref)
Low discrimination	13.1 (497)	2.06 (1.78,2.44)	2.08 (1.78,2.44)
Moderate/high discrimination	23.0 (200)	3.60 (3.11,4.49)	3.74 (3.11,4.49)
**Outcome: high or very high psychological distress (N=7656)* †**			
No discrimination	24.9 (785)	1 (Ref)	1 (Ref)
Low discrimination	45.1 (1651)	1.81 (1.66,1.92)	1.78 (1.66,1.92)
Moderate/high discrimination	63.0 (524)	2.53 (2.29,2.69)	2.48 (2.29,2.69)
**Outcome: doctor-diagnosed anxiety (N=8100)* †**			
No discrimination	22.5 (756)	1 (Ref)	1 (Ref)
Low discrimination	33.3 (1280)	1.48 (1.30,1.52)	1.41 (1.30,1.52)
Moderate/high discrimination	37.1 (331)	1.65 (1.44,1.78)	1.60 (1.44,1.78)
**Outcome: doctor-diagnosed depression (N=8100)* †**			
No discrimination	26.7 (898)	1 (Ref)	1 (Ref)
Low discrimination	37.8 (1456)	1.42 (1.31,1.51)	1.41 (1.31,1.51)
Moderate/high discrimination	42.4 (378)	1.59 (1.49,1.79)	1.63 (1.49,1.79)
**CULTURE AND IDENTITY OUTCOMES**			
**Outcome: low control over life (N=7929)* †**			
No discrimination	9.2 (304)	1 (Ref)	1 (Ref)
Low discrimination	22.1 (831)	2.39 (2.03,2.61)	2.30 (2.03,2.61)
Moderate/high discrimination	37.3 (323)	4.04 (3.27,4.34)	3.77 (3.27,4.34)
**Outcome: torn between cultures (N=7620)* †**			
No discrimination	35.7 (1124)	1 (Ref)	1 (Ref)
Low discrimination	59.0 (2148)	1.65 (1.51,1.69)	1.60 (1.51,1.69)
Moderate/high discrimination	63.7 (527)	1.79 (1.58,1.82)	1.69 (1.58,1.82)
**Outcome: disconnected from Aboriginal and/or Torres Strait Islander culture (N=7378)* †**			
No discrimination	63.4 (1903)	1 (Ref)	1 (Ref)
Low discrimination	73.4 (2617)	1.16 (1.08,1.15)	1.12 (1.08,1.15)
Moderate/high discrimination	71.1 (575)	1.12 (1.03,1.14)	1.08 (1.03,1.14)
**Outcome: choose not to self-identify as Aboriginal and/or Torres Strait Islander (N=8100)* †**			

No discrimination	8.9 (299)	1 (Ref)	1 (Ref)
Low discrimination	13.0 (501)	1.46 (1.25,1.65)	1.44 (1.25,1.65)
Moderate/high discrimination	16.4 (146)	1.84 (1.47,2.14)	1.77 (1.47,2.14)
**HEALTH BEHAVIOUR OUTCOMES**			
**Outcome: currently smoke (N=7992)* †**			
No discrimination	17.4 (576)	1 (Ref)	1 (Ref)
Low discrimination	27.8 (1058)	1.60 (1.35,1.62)	1.48 (1.35,1.62)
Moderate/high discrimination	45.2 (396)	2.60 (1.99,2.46)	2.21 (1.99,2.46)
**Outcome: gambled in last year (N=7934)* †**			
No discrimination	42.4 (1403)	1 (Ref)	1 (Ref)
Low discrimination	47.0 (1764)	1.11 (1.07,1.19)	1.12 (1.07,1.19)
Moderate/high discrimination	46.6 (404)	1.10 (1.02,1.20)	1.10 (1.02,1.20)
**Outcome: ever shaky if don't drink alcohol (N=7804)* †**			
No discrimination	2.2 (71)	1 (Ref)	1 (Ref)
Low discrimination	4.7 (172)	2.13 (1.73,3.00)	2.28 (1.73,3.00)
Moderate/high discrimination	11.0 (93)	5.02 (3.64,6.76)	4.96 (3.64,6.76)
**HEALTH OUTCOMES**			
**Outcome: poor/fair general health (N=7977)* †**			
No discrimination	24.5 (811)	1 (Ref)	1 (Ref)
Low discrimination	32.5 (1230)	1.33 (1.36,1.58)	1.46 (1.36,1.58)
Moderate/high discrimination	46.3 (405)	1.89 (1.97,2.37)	2.16 (1.97,2.37)
**Outcome: doctor-diagnosed heart disease (N=8100)* † ‡**			
No discrimination	11.4 (384)	1 (Ref)	1 (Ref)
Low discrimination	10.2 (391)	0.89 (1.03,1.33)	1.17 (1.03,1.33)
Moderate/high discrimination	10.8 (96)	0.94 (1.22,1.85)	1.50 (1.22,1.85)
**Outcome: doctor-diagnosed high cholesterol (N=8100)* †**			
No discrimination	29.9 (1006)	1 (Ref)	1 (Ref)
Low discrimination	26.1 (1004)	0.87 (1.00,1.15)	1.07 (1.00,1.15)
Moderate/high discrimination	24.2 (216)	0.81 (1.02,1.29)	1.15 (1.02,1.29)
**Outcome: doctor-diagnosed high blood pressure (N=8100)* † ‡**			
No discrimination	34.2 (1148)	1 (Ref)	1 (Ref)
Low discrimination	30.3 (1166)	0.89 (1.00,1.12)	1.06 (1.00,1.12)
Moderate/high discrimination	31.1 (277)	0.91 (1.09,1.32)	1.20 (1.09,1.32)
**Outcome: doctor-diagnosed diabetes (N=8100)* †**			
No discrimination	15.7 (528)	1 (Ref)	1 (Ref)
Low discrimination	17.4 (671)	1.11 (1.17,1.43)	1.29 (1.17,1.43)
Moderate/high discrimination	18.4 (164)	1.17 (1.30,1.78)	1.52 (1.30,1.78)

The adjusted models are adjusted for age group, gender (male or female), and remoteness. Due to small numbers, participants identifying as another gender are excluded from all regression analyses as the fully-adjusted models are adjusted for gender. All models exclude participants missing the outcome of interest.

* Indicates that the overall discrimination score variable is significant in the adjusted model, with the p-value for the Wald test <0.05.

† Indicates that the trend is significant in the adjusted model (when discrimination score is included as a continuous variable, set to the mean total score for each discrimination category), with the p-value for trend <0.05.

‡ Indicates that the adjusted model for the outcome (heart disease, blood pressure) employs a collapsed age categorisation (16-65 years, ≥66 years) due to small cells.

Table S3. Relationship between experiences of everyday discrimination, with and without attribution to Indigeneity, and wellbeing outcomes (N=up to 8039)

	% (n) with outcome	Unadjusted PR (95%CI)	Adjusted PR (95%CI)
**SEWB OUTCOMES**			
**Outcome: frequent experience of pain (N=7759) ***			
No discrimination	37.4 (1210)	1 (Ref)	1 (Ref)
Low discrimination			
without attribution	52.4 (705)	1.40 (1.35,1.55)	1.45 (1.35,1.55)
with attribution	42.7 (996)	1.14 (1.13,1.28)	1.20 (1.13,1.28)
Mod/high discrimination			
without attribution	56.3 (45)	1.51 (1.38,2.02)	1.67 (1.38,2.02)
with attribution	54.2 (412)	1.45 (1.43,1.68)	1.55 (1.43,1.68)
**Outcome: low life satisfaction (N=7874) ***			
No discrimination	15.0 (495)	1 (Ref)	1 (Ref)
Low discrimination			
without attribution	31.9 (435)	2.12 (1.88,2.35)	2.10 (1.88,2.35)
with attribution	30.3 (715)	2.02 (1.78,2.19)	1.98 (1.78,2.19)
Mod/high discrimination			
without attribution	56.4 (44)	3.75 (3.06,4.68)	3.78 (3.06,4.68)
with attribution	52.6 (411)	3.50 (3.07,3.80)	3.41 (3.07,3.80)
**Outcome: low happiness (N=7891) ***			
No discrimination	6.4 (210)	1 (Ref)	1 (Ref)
Low discrimination			
without attribution	14.3 (196)	2.24 (1.88,2.72)	2.26 (1.88,2.72)
with attribution	12.4 (294)	1.95 (1.67,2.35)	1.98 (1.67,2.35)
Mod/high discrimination			
without attribution	25.9 (21)	4.07 (3.13,6.79)	4.61 (3.13,6.79)
with attribution	22.5 (175)	3.52 (3.02,4.41)	3.65 (3.02,4.41)
**Outcome: high or very high psychological distress (N=7602) ***			
No discrimination	24.9 (785)	1 (Ref)	1 (Ref)
Low discrimination			
without attribution	44.9 (595)	1.81 (1.64,1.95)	1.79 (1.64,1.95)
with attribution	44.9 (1030)	1.81 (1.64,1.91)	1.77 (1.64,1.91)
Mod/high discrimination			
without attribution	62.3 (48)	2.51 (2.09,3.01)	2.51 (2.09,3.01)
with attribution	62.9 (469)	2.53 (2.28,2.69)	2.47 (2.28,2.69)
**Outcome: doctor-diagnosed anxiety (N=8039) ***			
No discrimination	22.5 (756)	1 (Ref)	1 (Ref)
Low discrimination			
without attribution	34.3 (476)	1.53 (1.35,1.62)	1.48 (1.35,1.62)
with attribution	32.6 (787)	1.45 (1.25,1.48)	1.36 (1.25,1.48)
Mod/high discrimination			
without attribution	36.1 (30)	1.61 (1.38,2.37)	1.81 (1.38,2.37)
with attribution	37.1 (296)	1.65 (1.41,1.75)	1.57 (1.41,1.75)
**Outcome: doctor-diagnosed depression (N=8039) ***			
No discrimination	26.7 (898)	1 (Ref)	1 (Ref)
Low discrimination			
without attribution	40.5 (562)	1.52 (1.39,1.64)	1.51 (1.39,1.64)
with attribution	36.2 (872)	1.35 (1.24,1.45)	1.34 (1.24,1.45)

Mod/high discrimination			
without attribution	37.4 (31)	1.40 (1.32,2.22)	1.71 (1.32,2.22)
with attribution	42.7 (341)	1.60 (1.47,1.78)	1.62 (1.47,1.78)
**CULTURE AND IDENTITY OUTCOMES**			
**Outcome: low control over life (N=7870) ***			
No discrimination	9.2 (304)	1 (Ref)	1 (Ref)
Low discrimination			
without attribution	23.5 (321)	2.55 (2.15,2.87)	2.48 (2.15,2.87)
with attribution	21.1 (497)	2.29 (1.91,2.50)	2.18 (1.91,2.50)
Mod/high discrimination			
without attribution	43.8 (35)	4.74 (3.40,5.89)	4.47 (3.40,5.89)
with attribution	36.7 (284)	3.97 (3.20,4.28)	3.70 (3.20,4.28)
**Outcome: torn between cultures (N=7563) ***			
No discrimination	35.7 (1124)	1 (Ref)	1 (Ref)
Low discrimination			
without attribution	52.7 (674)	1.48 (1.35,1.55)	1.45 (1.35,1.55)
with attribution	62.7 (1451)	1.76 (1.60,1.79)	1.69 (1.60,1.79)
Mod/high discrimination			
without attribution	55.9 (38)	1.57 (1.21,1.89)	1.51 (1.21,1.89)
with attribution	64.3 (483)	1.80 (1.59,1.84)	1.71 (1.59,1.84)
**Outcome: disconnected from Aboriginal and/or Torres Strait Islander culture (N=7323) ***			
No discrimination	63.4 (1903)	1 (Ref)	1 (Ref)
Low discrimination			
without attribution	75.0 (940)	1.18 (1.11,1.20)	1.15 (1.11,1.20)
with attribution	72.8 (1649)	1.15 (1.06,1.14)	1.10 (1.06,1.14)
Mod/high discrimination			
without attribution	68.3 (43)	1.08 (0.91,1.27)	1.07 (0.91,1.27)
with attribution	71.1 (525)	1.12 (1.03,1.13)	1.08 (1.03,1.13)
**Outcome: choose not to self-identify as Aboriginal and/or Torres Strait Islander (N=8039) ***			
No discrimination	8.9 (299)	1 (Ref)	1 (Ref)
Low discrimination			
without attribution	13.4 (186)	1.51 (1.24,1.76)	1.48 (1.24,1.76)
with attribution	12.8 (309)	1.44 (1.21,1.64)	1.40 (1.21,1.64)
Mod/high discrimination			
without attribution	15.7 (13)	1.76 (1.03,2.88)	1.72 (1.03,2.88)
with attribution	16.0 (128)	1.80 (1.41,2.10)	1.72 (1.41,2.10)
**HEALTH BEHAVIOUR OUTCOMES**			
**Outcome: currently smoke (N=7932) ***			
No discrimination	17.4 (576)	1 (Ref)	1 (Ref)
Low discrimination			
without attribution	27.0 (370)	1.55 (1.32,1.66)	1.48 (1.32,1.66)
with attribution	28.2 (672)	1.62 (1.34,1.63)	1.47 (1.34,1.63)
Mod/high discrimination			
without attribution	42.0 (34)	2.41 (1.37,2.35)	1.80 (1.37,2.35)
with attribution	45.4 (356)	2.61 (2.03,2.52)	2.26 (2.03,2.52)
**Outcome: gambled in last year (N=7877) ***			
No discrimination	42.4 (1403)	1 (Ref)	1 (Ref)
Low discrimination			
without attribution	47.5 (645)	1.12 (1.05,1.20)	1.13 (1.05,1.20)

with attribution	46.8 (1102)	1.10 (1.06,1.20)	1.13 (1.06,1.20)
Mod/high discrimination			
without attribution	28.8 (23)	0.68 (0.50,1.00)	0.71 (0.50,1.00)
with attribution	48.4 (376)	1.14 (1.05,1.24)	1.14 (1.05,1.24)
**Outcome: ever shaky if don't drink alcohol (N=7749) ***			
No discrimination	2.2 (71)	1 (Ref)	1 (Ref)
Low discrimination			
without attribution	4.1 (55)	1.89 (1.38,2.75)	1.95 (1.38,2.75)
with attribution	5.0 (115)	2.28 (1.86,3.35)	2.50 (1.86,3.35)
Mod/high discrimination			
without attribution	7.7 (6)	3.53 (1.44,7.26)	3.24 (1.44,7.26)
with attribution	11.3 (86)	5.17 (3.76,7.03)	5.14 (3.76,7.03)
**HEALTH OUTCOMES**			
**Outcome: poor/fair general health (N=7918) ***			
No discrimination	24.5 (811)	1 (Ref)	1 (Ref)
Low discrimination			
without attribution	35.3 (484)	1.44 (1.41,1.69)	1.54 (1.41,1.69)
with attribution	30.7 (727)	1.25 (1.29,1.53)	1.41 (1.29,1.53)
Mod/high discrimination			
without attribution	46.9 (38)	1.92 (1.91,2.96)	2.38 (1.91,2.96)
with attribution	46.0 (360)	1.88 (1.94,2.35)	2.13 (1.94,2.35)
**Outcome: doctor-diagnosed heart disease (N=8039) * †**			
No discrimination	11.4 (384)	1 (Ref)	1 (Ref)
Low discrimination			
without attribution	11.5 (160)	1.01 (1.01,1.42)	1.20 (1.01,1.42)
with attribution	9.4 (226)	0.82 (1.00,1.35)	1.16 (1.00,1.35)
Mod/high discrimination			
without attribution	8.4 (7)	0.74 (0.63,2.52)	1.26 (0.63,2.52)
with attribution	10.9 (87)	0.95 (1.23,1.88)	1.52 (1.23,1.88)
**Outcome: doctor-diagnosed high cholesterol (N=8039) ***			
No discrimination	29.9 (1006)	1 (Ref)	1 (Ref)
Low discrimination			
without attribution	29.1 (403)	0.97 (1.01,1.20)	1.10 (1.01,1.20)
with attribution	24.2 (584)	0.81 (0.96,1.13)	1.04 (0.96,1.13)
Mod/high discrimination			
without attribution	18.1 (15)	0.60 (0.61,1.46)	0.94 (0.61,1.46)
with attribution	24.9 (199)	0.83 (1.04,1.33)	1.17 (1.04,1.33)
**Outcome: doctor-diagnosed high blood pressure (N=8039) * †**			
No discrimination	34.2 (1148)	1 (Ref)	1 (Ref)
Low discrimination			
without attribution	33.4 (463)	0.98 (1.00,1.17)	1.08 (1.00,1.17)
with attribution	28.5 (686)	0.83 (0.97,1.12)	1.04 (0.97,1.12)
Mod/high discrimination			
without attribution	21.7 (18)	0.63 (0.61,1.33)	0.90 (0.61,1.33)
with attribution	32.0 (255)	0.94 (1.12,1.37)	1.24 (1.12,1.37)
**Outcome: doctor-diagnosed diabetes (N=8039) ***			
No discrimination	15.7 (528)	1 (Ref)	1 (Ref)
Low discrimination			
without attribution	17.2 (239)	1.10 (1.06,1.39)	1.21 (1.06,1.39)

with attribution	17.4 (420)	1.11 (1.20,1.50)	1.34 (1.20,1.50)
Mod/high discrimination			
without attribution	12.1 (10)	0.77 (0.60,1.92)	1.08 (0.60,1.92)
with attribution	19.2 (153)	1.22 (1.34,1.85)	1.57 (1.34,1.85)

The adjusted models are adjusted for age group, gender (male or female), and remoteness. Due to small numbers, participants identifying as another gender are excluded from all regression analyses as the models are adjusted for gender. All models exclude participants missing the outcome of interest.

* Indicates that the overall discrimination score variable is significant in the adjusted model, with the p-value for the Wald test <0.05.

† Indicates that the adjusted model for the outcome (heart disease, blood pressure) employs a collapsed age categorisation (16-65 years, ≥66 years) due to small cells.

Table S4. Relationship between experiences of everyday discrimination and wellbeing outcomes, additionally adjusted for education (N=up to 8017) and for financial status (N=up to 7865)

	PR (95%CI) additionally adjusted for education	PR (95%CI) additionally adjusted for financial situation
**SEWB OUTCOMES**		
**Outcome: frequent experience of pain* †**		
No discrimination	1 (Ref)	1 (Ref)
Low discrimination	1.34 (1.27,1.41)	1.20 (1.13,1.27)
Moderate/high discrimination	1.60 (1.49,1.73)	1.32 (1.23,1.43)
**Outcome: low life satisfaction* †**		
No discrimination	1 (Ref)	1 (Ref)
Low discrimination	2.02 (1.84,2.22)	1.61 (1.46,1.76)
Moderate/high discrimination	3.26 (2.93,3.63)	2.18 (1.96,2.42)
**Outcome: low happiness* †**		
No discrimination	1 (Ref)	1 (Ref)
Low discrimination	2.11 (1.80,2.47)	1.66 (1.41,1.94)
Moderate/high discrimination	3.73 (3.10,4.48)	2.39 (1.97,2.89)
**Outcome: high or very high psychological distress* †**		
No discrimination	1 (Ref)	1 (Ref)
Low discrimination	1.79 (1.67,1.92)	1.56 (1.45,1.67)
Moderate/high discrimination	2.40 (2.21,2.61)	1.87 (1.72,2.03)
**Outcome: doctor-diagnosed anxiety* †**		
No discrimination	1 (Ref)	1 (Ref)
Low discrimination	1.42 (1.31,1.53)	1.34 (1.24,1.45)
Moderate/high discrimination	1.64 (1.47,1.82)	1.43 (1.28,1.60)
**Outcome: doctor-diagnosed depression* †**		
No discrimination	1 (Ref)	1 (Ref)
Low discrimination	1.42 (1.32,1.52)	1.30 (1.21,1.40)
Moderate/high discrimination	1.66 (1.51,1.82)	1.35 (1.22,1.49)
**CULTURE AND IDENTITY OUTCOMES**		
**Outcome: low control over life* †**		
No discrimination	1 (Ref)	1 (Ref)
Low discrimination	2.29 (2.02,2.60)	1.83 (1.61,2.07)
Moderate/high discrimination	3.66 (3.17,4.22)	2.42 (2.10,2.80)
**Outcome: torn between cultures* †**		
No discrimination	1 (Ref)	1 (Ref)
Low discrimination	1.58 (1.50,1.67)	1.61 (1.52,1.70)
Moderate/high discrimination	1.72 (1.60,1.84)	1.70 (1.58,1.83)
**Outcome: disconnected from Aboriginal and/or Torres Strait Islander culture* †**		
No discrimination	1 (Ref)	1 (Ref)
Low discrimination	1.11 (1.07,1.14)	1.12 (1.08,1.15)
Moderate/high discrimination	1.09 (1.04,1.14)	1.08 (1.03,1.14)
**Outcome: choose not to self-identify as Aboriginal and/or Torres Strait Islander* †**		
No discrimination	1 (Ref)	1 (Ref)
Low discrimination	1.43 (1.24,1.64)	1.39 (1.21,1.60)
Moderate/high discrimination	1.86 (1.54,2.25)	1.69 (1.38,2.06)
**HEALTH BEHAVIOUR OUTCOMES**		
**Outcome: currently smoke* †**		
No discrimination	1 (Ref)	1 (Ref)
Low discrimination	1.49 (1.37,1.63)	1.29 (1.18,1.42)
Moderate/high discrimination	2.01 (1.81,2.23)	1.66 (1.49,1.86)
**Outcome: gambled in last year* †**		
No discrimination	1 (Ref)	1 (Ref)
Low discrimination	1.13 (1.07,1.19)	1.12 (1.06,1.18)
Moderate/high discrimination	1.11 (1.03,1.21)	1.12 (1.03,1.22)
**Outcome: ever shaky if don’t drink alcohol* †**		
No discrimination	1 (Ref)	1 (Ref)
Low discrimination	2.29 (1.74,3.01)	1.87 (1.41,2.49)
Moderate/high discrimination	4.47 (3.27,6.12)	3.47 (2.50,4.80)
**HEALTH OUTCOMES**		
**Outcome: poor/fair general health* †**		
No discrimination	1 (Ref)	1 (Ref)
Low discrimination	1.46 (1.36,1.57)	1.22 (1.13,1.31)
Moderate/high discrimination	2.02 (1.85,2.21)	1.52 (1.39,1.67)
**Outcome: doctor-diagnosed heart disease* †**		
No discrimination	1 (Ref)	1 (Ref)
Low discrimination	1.16 (1.02,1.32)	1.08 (0.95,1.24)
Moderate/high discrimination	1.47 (1.20,1.81)	1.34 (1.08,1.66)
**Outcome: doctor-diagnosed high cholesterol***		
No discrimination	1 (Ref)	1 (Ref)
Low discrimination	1.07 (1.00,1.15)	1.05 (0.98,1.13)
Moderate/high discrimination	1.14 (1.01,1.28)	1.11 (0.98,1.25)

**Outcome: doctor-diagnosed high blood pressure* †**		
No discrimination	1 (Ref)	1 (Ref)
Low discrimination	1.06 (0.99,1.12)	1.04 (0.98,1.11)
Moderate/high discrimination	1.19 (1.08,1.32)	1.18 (1.06,1.30)
**Outcome: doctor-diagnosed diabetes* †**		
No discrimination	1 (Ref)	1 (Ref)
Low discrimination	1.30 (1.18,1.44)	1.17 (1.06,1.30)
Moderate/high discrimination	1.50 (1.28,1.75)	1.29 (1.09,1.52)

The adjusted models are adjusted for age group, gender (male or female), remoteness, and education or financial situation, as indicated. Due to small numbers, participants identifying as another gender are excluded from all regression analyses as the fully-adjusted models are adjusted for gender. All models exclude participants missing the outcome of interest.

* Indicates that the overall discrimination score variable is significant in the model for education, with the p-value for the Wald test <0.05.

† Indicates that the overall discrimination score variable is significant in the model for financial situation, with the p-value for the Wald test <0.05.

Table S5. Relationship between experiences of everyday discrimination, with and without attribution to Indigeneity, and wellbeing outcomes, additionally adjusted for education (N=up to 7957) and for financial status (N=up to 7808)

	PR (95%CI) additionally adjusted for education	PR (95%CI) additionally adjusted for financial situation
**SEWB OUTCOMES**		
**Outcome: frequent experience of pain* † ‡ §**		
No discrimination	1 (Ref)	1 (Ref)
Low discrimination		
without attribution	1.44 (1.34,1.53)	1.29 (1.21,1.38)
with attribution	1.21 (1.14,1.29)	1.10 (1.03,1.17)
Mod/high discrimination		
without attribution	1.59 (1.31,1.94)	1.29 (1.03,1.60)
with attribution	1.49 (1.37,1.61)	1.28 (1.17,1.39)
**Outcome: low life satisfaction* †**		
No discrimination	1 (Ref)	1 (Ref)
Low discrimination		
without attribution	2.07 (1.85,2.31)	1.66 (1.48,1.85)
with attribution	1.99 (1.79,2.20)	1.57 (1.42,1.74)
Mod/high discrimination		
without attribution	3.53 (2.85,4.37)	2.44 (2.02,2.94)
with attribution	3.24 (2.91,3.61)	2.17 (1.95,2.42)
**Outcome: low happiness* †**		
No discrimination	1 (Ref)	1 (Ref)
Low discrimination		
without attribution	2.29 (1.90,2.75)	1.78 (1.48,2.15)
with attribution	2.01 (1.69,2.39)	1.58 (1.33,1.88)
Mod/high discrimination		
without attribution	4.67 (3.17,6.87)	3.00 (2.01,4.46)
with attribution	3.63 (3.00,4.39)	2.33 (1.91,2.84)
**Outcome: high or very high psychological distress* †**		
No discrimination	1 (Ref)	1 (Ref)
Low discrimination		
without attribution	1.78 (1.63,1.94)	1.56 (1.44,1.70)
with attribution	1.79 (1.66,1.93)	1.54 (1.43,1.67)
Mod/high discrimination		
without attribution	2.37 (1.95,2.87)	1.75 (1.43,2.14)
with attribution	2.40 (2.20,2.61)	1.87 (1.72,2.04)
**Outcome: doctor-diagnosed anxiety* † ‡ §**		
No discrimination	1 (Ref)	1 (Ref)
Low discrimination		
without attribution	1.48 (1.35,1.62)	1.42 (1.29,1.56)
with attribution	1.35 (1.25,1.47)	1.28 (1.17,1.39)
Mod/high discrimination		
without attribution	1.88 (1.44,2.46)	1.74 (1.34,2.25)
with attribution	1.59 (1.43,1.77)	1.38 (1.23,1.54)
**Outcome: doctor-diagnosed depression* † §**		
No discrimination	1 (Ref)	1 (Ref)
Low discrimination		
without attribution	1.52 (1.40,1.66)	1.37 (1.26,1.49)
with attribution	1.35 (1.25,1.45)	1.20 (1.11,1.29)
Mod/high discrimination		
without attribution	1.79 (1.38,2.31)	1.49 (1.16,1.92)
with attribution	1.64 (1.49,1.81)	1.28 (1.15,1.41)
**CULTURE AND IDENTITY OUTCOMES**		
**Outcome: low control over life* † §**		
No discrimination	1 (Ref)	1 (Ref)
Low discrimination		
without attribution	2.45 (2.12,2.83)	1.93 (1.67,2.23)
with attribution	2.18 (1.91,2.50)	1.74 (1.52,1.99)
Mod/high discrimination		
without attribution	4.39 (3.34,5.77)	3.02 (2.35,3.86)
with attribution	3.58 (3.09,4.14)	2.35 (2.03,2.72)
**Outcome: torn between cultures* †**		
No discrimination	1 (Ref)	1 (Ref)
Low discrimination		
without attribution	1.44 (1.34,1.54)	1.46 (1.36,1.57)
with attribution	1.66 (1.57,1.76)	1.69 (1.60,1.79)
Mod/high discrimination		
without attribution	1.50 (1.19,1.88)	1.48 (1.17,1.89)
with attribution	1.74 (1.62,1.87)	1.72 (1.60,1.85)
**Outcome: disconnected from Aboriginal and/or Torres Strait Islander culture* †**		

No discrimination	1 (Ref)	1 (Ref)
Low discrimination		
without attribution	1.16 (1.12,1.20)	1.16 (1.11,1.20)
with attribution	1.08 (1.05,1.12)	1.10 (1.06,1.14)
Mod/high discrimination		
without attribution	1.07 (0.90,1.26)	1.08 (0.92,1.28)
with attribution	1.08 (1.03,1.14)	1.08 (1.02,1.13)
**Outcome: choose not to self-identify as Aboriginal and/or Torres Strait Islander* †**		
No discrimination	1 (Ref)	1 (Ref)
Low discrimination		
without attribution	1.48 (1.24,1.76)	1.43 (1.19,1.70)
with attribution	1.39 (1.19,1.62)	1.37 (1.17,1.60)
Mod/high discrimination		
without attribution	1.85 (1.11,3.10)	1.48 (0.82,2.67)
with attribution	1.81 (1.48,2.20)	1.67 (1.35,2.05)
**HEALTH BEHAVIOUR OUTCOMES**		
**Outcome: currently smoke* † ‡ §**		
No discrimination	1 (Ref)	1 (Ref)
Low discrimination		
without attribution	1.44 (1.29,1.61)	1.27 (1.13,1.42)
with attribution	1.52 (1.38,1.68)	1.30 (1.18,1.44)
Mod/high discrimination		
without attribution	1.67 (1.28,2.17)	1.41 (1.07,1.86)
with attribution	2.12 (1.90,2.35)	1.70 (1.52,1.90)
**Outcome: gambled in last year* †**		
No discrimination	1 (Ref)	1 (Ref)
Low discrimination		
without attribution	1.13 (1.06,1.21)	1.12 (1.04,1.20)
with attribution	1.13 (1.07,1.20)	1.13 (1.06,1.20)
Mod/high discrimination		
without attribution	0.67 (0.47,0.97)	0.74 (0.52,1.06)
with attribution	1.15 (1.06,1.25)	1.15 (1.05,1.25)
**Outcome: ever shaky if don't drink alcohol* † §**		
No discrimination	1 (Ref)	1 (Ref)
Low discrimination		
without attribution	1.88 (1.33,2.66)	1.48 (1.03,2.12)
with attribution	2.58 (1.92,3.45)	1.88 (1.39,2.55)
Mod/high discrimination		
without attribution	2.88 (1.27,6.50)	1.81 (0.80,4.09)
with attribution	4.65 (3.39,6.39)	3.15 (2.27,4.36)
**HEALTH OUTCOMES**		
**Outcome: poor/fair general health***		
No discrimination	1 (Ref)	--
Low discrimination		
without attribution	1.51 (1.39,1.65)	--
with attribution	1.42 (1.31,1.54)	--
Mod/high discrimination		
without attribution	2.22 (1.78,2.76)	--
with attribution	1.99 (1.82,2.19)	--
**Outcome: doctor-diagnosed heart disease***		
No discrimination	1 (Ref)	1 (Ref)
Low discrimination		
without attribution	1.19 (1.01,1.41)	1.09 (0.92,1.30)
with attribution	1.15 (0.99,1.34)	1.08 (0.92,1.26)
Mod/high discrimination		
without attribution	1.27 (0.63,2.56)	1.14 (0.53,2.43)
with attribution	1.49 (1.20,1.84)	1.35 (1.08,1.68)
**Outcome: doctor-diagnosed high cholesterol***		
No discrimination	1 (Ref)	1 (Ref)
Low discrimination		
without attribution	1.10 (1.01,1.21)	1.08 (0.99,1.18)
with attribution	1.04 (0.96,1.13)	1.02 (0.94,1.11)
Mod/high discrimination		
without attribution	0.91 (0.58,1.42)	1.01 (0.65,1.56)
with attribution	1.17 (1.04,1.32)	1.12 (0.98,1.27)
**Outcome: doctor-diagnosed high blood pressure* †**		
No discrimination	1 (Ref)	1 (Ref)
Low discrimination		
without attribution	1.08 (0.99,1.16)	1.07 (0.98,1.16)
with attribution	1.04 (0.97,1.12)	1.03 (0.96,1.11)
Mod/high discrimination		
without attribution	0.91 (0.62,1.35)	1.03 (0.71,1.50)
with attribution	1.23 (1.11,1.36)	1.19 (1.07,1.32)
**Outcome: doctor-diagnosed diabetes* †**		

No discrimination	1 (Ref)	1 (Ref)
Low discrimination		
without attribution	1.21 (1.06,1.39)	1.09 (0.95,1.25)
with attribution	1.36 (1.21,1.52)	1.22 (1.09,1.38)
Mod/high discrimination		
without attribution	1.09 (0.61,1.94)	0.95 (0.52,1.75)
with attribution	1.55 (1.32,1.82)	1.32 (1.12,1.56)

The adjusted models are adjusted for age group, gender (male or female), remoteness, and education or financial situation, as indicated. Due to small numbers, participants identifying as another gender are excluded from all regression analyses as the fully-adjusted models are adjusted for gender. All models exclude participants missing the outcome of interest.

* Indicates that the overall discrimination score variable is significant in the model for education, with the p-value for the Wald test <0.05.

† Indicates that the overall discrimination score variable is significant in the model for financial situation, with the p-value for the Wald test <0.05.

‡Indicates that the model adjusted for education employs a collapsed age categorisation (16-65 years, ≥66 years) due to small cells.

§ Indicates that the model adjusted for financial situation employs a collapsed age categorisation (16-65 years, ≥66 years) due to small cells.

-- Indicates that the model did not converge, even when the collapsed age categorisation was used.

References

1. Milnes, T.; Brinckley, M.; Calabria, B.; Olsen, A.; Lovett, R.; Thurber, K. Exploring Experiences of Pain in a Social and Emotional Wellbeing (SEWB) Context using data from Mayi Kuwayu: The National Study of Aboriginal and Torres Strait Islander Wellbeing. **(In Preparation)**.
2. Brinckley, M.-M.; Calabria, B.; Walker, J.; Thurber, K.A.; Lovett, R. Reliability, Validity, and Clinical Utility of a Culturally Modified Kessler Scale (MK-K5) in the Aboriginal and Torres Strait Islander Population. *BMC Public Health* **2021**, *21*, 1-15.
3. Reilly, R.E.; Doyle, J.; Bretherton, D.; Rowley, K.G.; Harvey, J.L.; Briggs, P.; Charles, S.; Calleja, J.; Patten, R.; Atkinson, V. Identifying psychosocial mediators of health amongst Indigenous Australians for the Heart Health Project. *Ethnicity & health* **2008**, *13*, 351-373, doi:10.1080/13557850801903046.
4. Santiago, P.H.R.; Nielsen, T.; Roberts, R.; Smithers, L.G.; Jamieson, L. Sense of personal control: Can it be assessed culturally unbiased across Aboriginal and non-Aboriginal Australians? *PloS one* **2020**, *15*, e0239384, doi:10.1371/journal.pone.0239384.
5. Weatherall, T.J.; Conigrave, K.M.; Conigrave, J.H.; Lee, K.K. What is the prevalence of current alcohol dependence and how is it measured for Indigenous people in Australia, New Zealand, Canada and the United States of America? A systematic review. *Addiction Science & Clinical Practice* **2020**, *15*, 1-11, doi:10.1186/s13722-020-00205-7.
